# Structural, optical, thermal and conducting properties of V_2−*x*_Li_*x*_O_5−*δ*_ (0.15 ≤ x ≤ 0.30) systems

**DOI:** 10.1038/s41598-020-57836-8

**Published:** 2020-01-23

**Authors:** Savidh Khan, K. Singh

**Affiliations:** School of Physics and Materials Science, Thapar Institute of Engineering & Technology, Patiala, 147004 India

**Keywords:** Batteries, Batteries, Fuel cells, Fuel cells

## Abstract

Lithium-doped vanadates (V_2−*x*_Li_*x*_O_5−*δ*_ (0.15 ≤ *x* ≤ 0.30)) are synthesized by melt-quench method. The physical, structural, optical, thermal and conducting properties of as-quenched samples are investigated using various experimental techniques to study their suitability for electrolyte in battery/solid oxide fuel cell application. X-ray diffraction (XRD) patterns confirm the formation of three different crystalline phases. FTIR and Raman spectra indicate that the doping of Li_2_O into V_2_O_5_ leads to a transition from VO_5_ into VO_4_ structural unit. The optical diffused reflectance spectra revealed that the optical band gap (*E*_*g*_) decreases from 2.2 to 2.08 eV while Urbach energy (*E*_*U*_) increases (0.31–0.41 eV) with the addition of Li_2_O content in place of vanadium. The thermal stability is studied by thermogravimetric analyser (TGA). The DC conductivity of the present samples is increased from 0.08 to 0.12 Scm^−1^ at 450 °C with Li_2_O doping. These materials can be used as electrolyte for battery/solid oxide fuel cell due to their good conductivity (~0.12 Scm^−1^) at 450 °C.

## Introduction

Solid oxide fuel cells (SOFCs) are most efficient electrochemical devices which convert chemical energy into electricity with heat and water as a by-product. SOFCs are considered as eco-friendly since there is no production of sulfur or nitrogen oxides (only CO_2_) along with fuel flexibility (H_2_, CO, hydrocarbons and biogases)^1–3^. Since, the high operating temperature of SOFC leads to degradation, coefficient of thermal expansion mismatch, electrode sintering and catalyst poisoning among the components of SOFCs^1–3^. Thus, a lot of research is carried out to develop new materials that can meet the requirements at lower operating temperature of SOFCs^3–5^. Doped V_2_O_5_ materials can be a good choice for low temperature (600–800 °C) SOFCs applications due to their good conductivity at lower temperature^6^. The electrical conduction in V_2_O_5_ based materials arises by exchanging of unpaired 3d^1^ electrons (hopping) between V^4+^ and V^5+^ valence states, V^4+^-O-V^5+^-O-V^4+^. These unpaired electrons are induced polarization around vanadium ions which leads to form polaron^6,7^. Alkali metal oxide doped vanadates have been studied widely due to their numerous applications in optical and electronic devices^1,2,8^. The chemical nature and concentration of dopants play a vital role to change the oxidation state of vanadium which leads to a creation of oxygen vacancies to maintain overall electrical neutrality of the vanadate systems^5,9,10^. Punia *et al*. have reported the increasing trend of density and molar volume due to change in structure from VO_4_ tetrahedral to VO_5_ trigonal bi-pyramid with the addition of Bi_2_O_3_ in zinc vanadates^11^. In addition to this, alkali ions doped materials usually show mixed electronic and ionic conductivity behavior with temperature. Doped bismuth vanadates (Bi_4_V_2_O_11−δ_) show good conductivity ~0.2 Scm^−1^ at 500 °C due to the higher oxygen vacancies and stabilization of high conducting γ-phase at room temperature^12^. The highest conductivity i.e. 6.6 × 10^−4^ Scm^−1^ for Bi_4_V_2−x_Al_x_O_11−δ_ (x = 0.2) system at 500 °C has been reported by Ravikant *et al*.^4^. Chakrabarty *et al.*^ 13^ studied the doping effect of Na_2_O on conducting properties of Na_2_O-V_2_O_5_ system and found an increment in conductivity (0.05 × 10^−3^–19.5 × 10^−3^ Scm^−1^) with Na_2_O concentration at room temperature. A study of V_2−x_Mg_x_O_5−δ_ (x = 0.05–0.30) systems has been revealed a decreasing trend in density (3.2–2.7 gcm^−3^) and thermal stability with MgO concentration. The structural transition has occurred from VO_5_ to VO_4_ polyhedra with MgO dopant. DC conductivity decreases from 10^−1^ Sm^−1^ to 10^−4^ Sm^−1^ while activation energy increases from 0.27 to 0.44 eV with an increase in MgO content in place of vanadium^6^. The optical bandgap increases whereas Urbach energy decreases with the increasing content of Bi_2_O_3_ due to decreasing oxygen vacancies^14^. Electrical properties of MnO_2_ doped V_2_O_5_ system are also reported^15^. Tsuzuki *et al*.^16^ have investigated V_2_O_5_-MO (M = Mg, Ca and Ba) systems and correlate the structural changes with electrical properties. In most of the cases, these doped systems are prepared by solid-state reaction/chemical method followed by slow cooling which leads to lower the conductivity due to ordering in oxygen vacancies^5^. It has been reported by some research groups that quenched samples have higher disordering/defects which leads to increase in the overall electrical conductivity. The high quenching rate encourages the reduction of vanadium which leads to higher disordering and hence may increase the overall conductivity^5,17^. Based on above discussion, it could be concluded that processing parameters and chemical nature of dopants affect the properties significantly particularly conductivity. Therefore, the motivation of the present work is to study the effect of systematic change of lower valance dopant (Li_2_O) concentration on optical, thermal and conducting properties of V_2_O_5_ grown by melt-quench technique. The V_2−*x*_Li_*x*_O_5−*δ*_ (*x* = 0.15–0.30) systems have been synthesized by melt-quench followed by various characterization techniques to study their structural, optical, thermal and electrical properties to check their suitability as electrolytes for SOFCs applications.

## Results and Discussion

### Physical properties

Various physical parameters such as density, molar volume and ionic concentration and their inter-ionic distance and polaron radius are calculated for the as-quenched samples and depicted in Table 1.

**Table 1:** Physical parameters with their labels of the as quenched samples.

Sample ID	Molecular weight, *M* (gmol^−1^)	Density, *ρ* (gcm^−3^)	Molar volume, *V*_*m*_ (cm^3^mol^−1^)	Ionic concentration, *N*_*i*_ (×10^23^ cm^−3^)	Inter-ionic distance, *R*_*i*_ (Ǻ)	Polaron radius, *r*_*p*_ (Ǻ)
VL-0.15	170.48	3.09	55.17	1.63	1.82	0.73
VL-0.20	166.68	3.00	55.56	2.16	1.66	0.67
VL-0.25	162.88	2.93	55.59	2.70	1.54	0.62
VL-0.30	159.08	2.86	55.62	3.24	1.45	0.58

**Figure 1:**
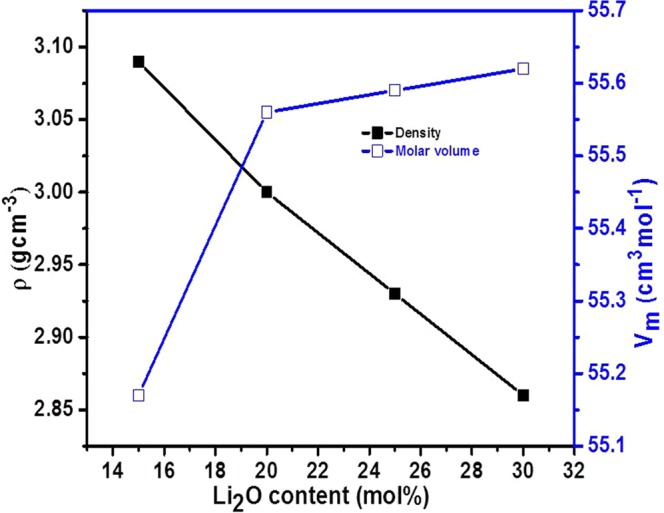
Change in density and molar volume with Li_2_O content in V_2_O_5_.

**Figure 2:**
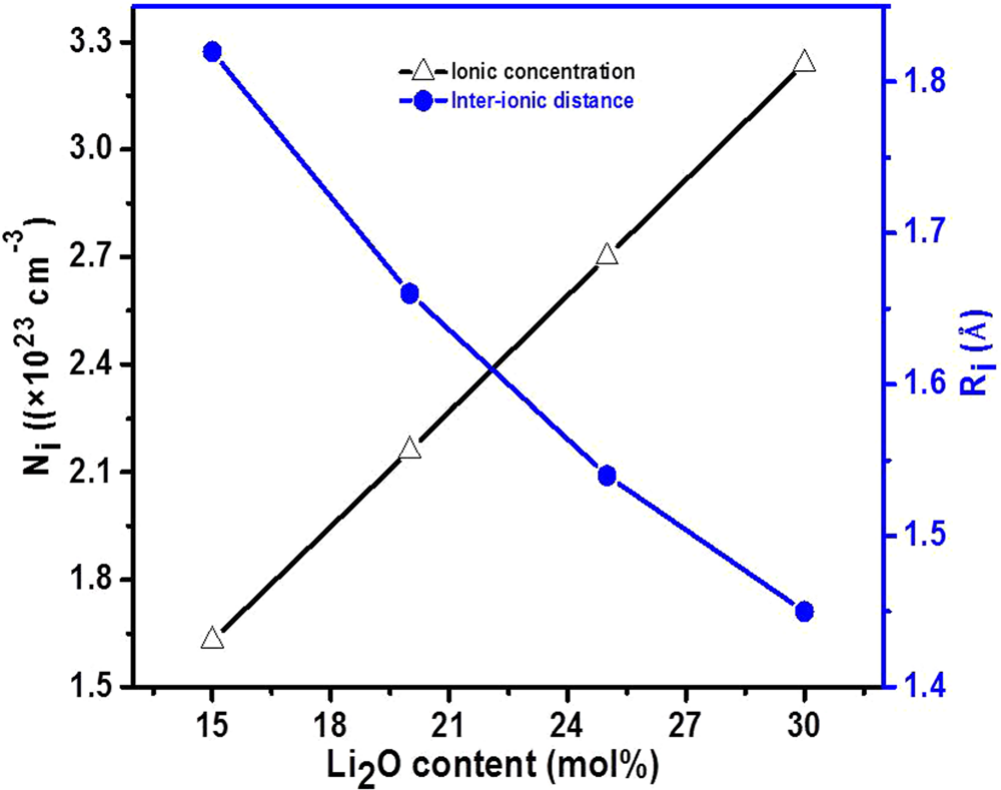
Change in ionic concentration and their inter-ionic distance with Li_2_O content in V_2_O_5_.

Figure 1 shows the variation of density (*ρ*) and molar volume (*V*_*m*_) with Li_2_O dopant of as-quenched samples. It is cleared (Fig. 1) that the density decreases while molar volume increases with increasing Li_2_O content. This decrement in the density is due to the replacement of the heavier V_2_O_5_ (3.36 gcm^−3^) by the lighter Li_2_O (2.01 gcm^−3^)^6,18^. On the other hand, the doping of Li^1+^ into V^5+^ also creates oxygen vacancies. The size of oxygen vacancy is smaller as compared to the size of the oxygen anion O^2−^. So, the volume of the samples with dopant also decreases which leads to increase in the density. However, a lower density of dopant Li_2_O as compared to parent V_2_O_5_ influences the density of samples more efficiently than decrease the volume due to creation of vacancies in the samples^16^. Hence, the density of samples decreases with the addition of dopant (Li_2_O) in place of vanadium. On the other hand, the increase in molar volume is ascribed to the rearrangement of the lattice which leads to creating oxygen vacancies due to VO_5_ → VO_4_ conversion with the substitution of Li^1+^ for V^5+^ (as discussed in FTIR and TGA section)^18^. This increase in molar volume supports that the Li_2_O enters the network and occupy the interstitial space, which leads to an increase in free volume. Figure 2 shows the change in ionic concentration (*Ni*) and their inter-ionic distance (*R*_*i*_) with Li_2_O concentration on the cost of vanadium for all the samples. The ionic concentration increases whereas inter-ionic distance decreases (1.8–1.4 Ǻ) with increasing Li_2_O content. Decrement in the inter-ionic distance might be increased the localization effect as also confirmed by the polarization radius values (Table 1). In general, the decrease in molar volume is responsible for the decrease in inter-ionic distance^18^. The polaron radius (*r*_*p*_) values are also calculated using *R*_*i*_ (Table 1). These polaron radius values decrease from 0.7 to 0.5 Ǻ (Table 1) with Li_2_O dopant due to increases *R*_*i*_.The magnitude of polaron radius values suggested that they are highly localized and this localization effect increases with dopant (Li_2_O) concentration.

### Structural properties

#### X-ray diffraction (XRD)

**Figure 3:**
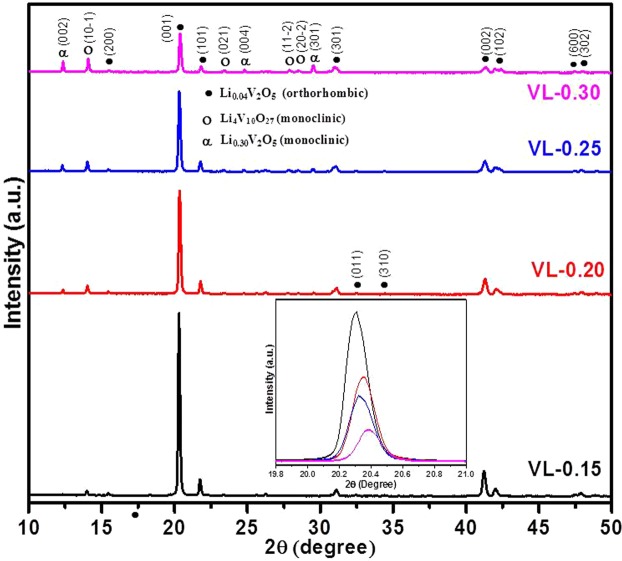
XRD patterns of Li_2_O doped V_2_O_5_ as-quenched samples. The inset shows the highest intensity peak shifting towards higher diffraction angle.

Figure 3 represents the XRD patterns of the Li_2_O modified V_2_O_5_ as-quenched samples. The as-quenched samples exhibit three crystalline phases i.e. major orthorhombic Li_0.04_V_2_O_5_ (ICDD: 01-085-0608), minor monoclinic Li_4_V_10_O_27_ (ICDD: 00-046-0187) and minor monoclinic Li_0.30_V_2_O_5_ (ICDD: 00-018-0755). The diffraction peak (001) at 20.3° corresponds to Li_0.04_V_2_O_5_ major phase, which shows higher angle shift with Li_2_O content as shown in the inset of Fig. 3. The shifting of peak towards higher diffraction angle side is the evidence of lattice contractions^19^. This is due to the smaller cationic radius Li^1+^ (0.167 nm) in comparison to V^5+^ (0.171 nm). As a result of the substitution of the larger cationic radius of V by the smaller cationic radius of Li, the lattice compression (compressive strain) arises in the Li_0.04_V_2_O_5_ crystalline phase^19^. Further comparing the peak intensities of the phases, it is observed that the intensity of the major phase (Li_0.04_V_2_O_5_) at 20.3° decreases whereas the intensity of the two other phases i.e. (Li_0.30_V_2_O_5_ and Li_4_V_10_O_27_) at 12.3° and 14.0° increases caused by doping of Li_2_O into the V_2_O_5_ lattice. So, the higher concentration of Li_2_O increases the volume fraction Li_0.30_V_2_O_5_ and Li_4_V_10_O_27_ phases as given in Table 2. So, the volume fraction of less symmetric phases (monoclinic) increases as dopant concentration increases.

**Table 2:** Optical and conductivity parameters of the as quenched samples.

Sample ID	*E*_*g*_ (eV)	*E*_*u*_ (eV)	*σ*_*ac*_ at 450 °C and 10^7^ Hz (Scm^−1^)	*σ*_*dc*_ at 450 °C (Scm^−1^)	*E*_*a*_ (eV) between 50–250 °C	Present phases	Volume fraction (%)
VL-0.15	2.22	0.31	0.11	0.08	0.26	Li_0.04_V_2_O_5_ Li_4_V_10_O_27_ Li_0.30_V_2_O_5_	95.35 3.05 1.58
VL-0.20	2.16	0.37	0.17	0.09	0.21	,,	86.45 8.30 5.10
VL-0.25	2.14	0.40	0.21	0.11	0.31	,,	80.74 11.97 7.27
VL-0.30	2.08	0.41	0.29	0.12	0.43	,,	60.26 20.70 18.00

#### FTIR spectroscopy analysis

**Figure 4:**
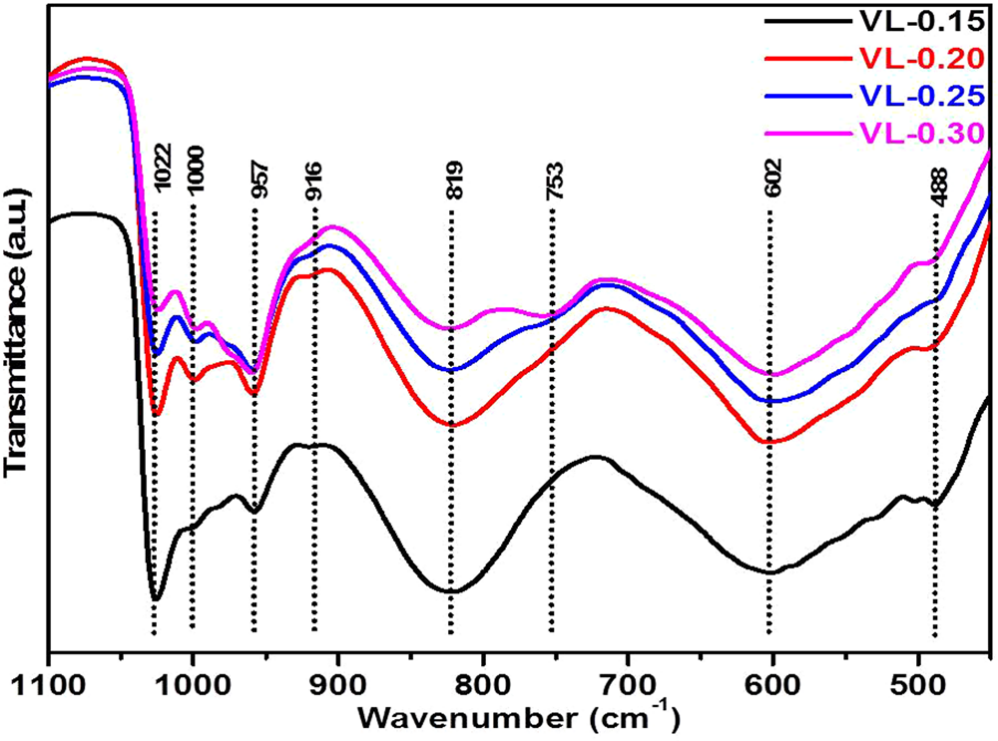
FTIR spectra of V_2−x_Li_x_O_5−δ_ (0.15 ≤ x ≤ 0.30) as-quenched samples.

**Figure 5:**
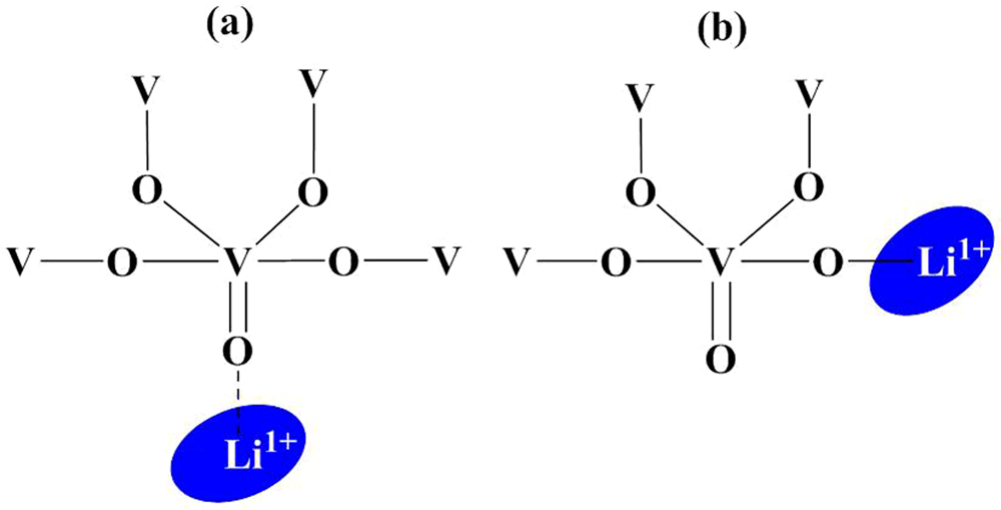
Schematic diagram of possible positions of the Li^1+^ ions into vanadium oxygen polyhedra: (**a**) interstitial sites; and (**b**) substitutional sites.

Figure 4 shows infrared (IR) spectra at room temperature to investigate the structural changes with increasing concentration of Li_2_O dopant. The IR spectra exhibited prominent absorption bands at 1022, 1000, 957, 916, 819, 753, 602, 488 cm^−1^. The absorption bands at 1022 and 1000 cm^−1^ are ascribed to the vibrations of isolated V=O vanadyl groups in VO_5_ trigonal bipyramids^20–22^. This characteristic stretching band with Li_2_O doping present samples, which is associated with the layer structure of the V_2_O_5_ ^20–23^. The broadness of this band decreases with increasing Li_2_O content due to the decrease in the number of ionic groups and V=O bonds. Additionally, this band has a small shift to the lower wavenumber, which suggests the reduction of vanadium (V^5+^ to V^4+^/V^3+^) due to the addition of lithium content^24^. The V–O bond length increases due to the larger size of V^4+^ (0.67 Ǻ) than V^5+^ (0.60 Ǻ), results in requiring lower energy for the lattice vibration^25^. The high-temperature melt quenching of the present samples could be responsible for the reduction in the oxidation states of vanadium due to scarcity of oxygen in the furnace at high temperature^24^. The band at 957 cm^−1^ assigned to the symmetric and anti-symmetric stretching vibration of the isolated VO_2_ groups in the VO_4_ polyhedra^20,26^. A small kink at 916 cm^−1^ has appeared with the addition of Li_2_O content. It is also assigned to the asymmetric stretching vibrations of the isolated VO_2_ groups in VO_4_ polyhedra^20,24^. Another band at about 819 cm^−1^ is attributed to the vibrations of isolated [VO_4_] tetrahedral. The broadness of this band decreases with Li_2_O content and splits into two bands (819 and 753 cm^−1^) at maximum Li_2_O content sample (VL-0.30). This new generated band is assigned to V–O–V stretching^6,20,26^. The broadening of the bands is directly related to the existence of different structural units of the same element with variable oxygens^6^. Thus, in the present case, the different structural units of vanadium decrease with the addition of Li_2_O content. The band at 602 cm^−1^ is ascribed to the bending vibrations of V–O–V units. The band at 488 cm^−1^ may be assigned to the bending modes of the V_2_O_5_ network consisting of [VO_5_] polyhedral and a characteristic vibration of Li^+^ cations^20,23,27^. This band shows a small shift to the higher wavenumber with the highest doping of Li_2_O (VL-0.30 sample), which shows that some portion of V^3+^/V^4+^ oxidized to V^5+^. The bond length between vanadium and oxygen decreases due to the smaller size of V^5+^ as compared to V^4+^/V^3+^, which leads to strengthening of this vibration^6,25,28^. According to Dimitriv *et al*.^26^, the vanadates can exist in the form of layers and chains and smaller complexes, which depends on the ratio of V_2_O_5_ and Li_2_O in the samples. The Li^1+^ ions can be situated between the vanadate chains, whereas some Li ions can be form independent polyhedra (LiO_n_), which can be located in the chains themselves^26^. This assumption suggests two different forms of Li ions distribution in the V–O polyhedra (interstitial sites or substitutional sites) as shown in Fig. 5(a,b). In interstitial condition, the Li ions can be located between vanadate chains and layers. Li ions can interact with the isolated V=O bonds, which leads to the longer bond length (Fig. 5(a)). In substitutional condition, Li ions can occupy positions in the vanadate chain, which leads to break the some of the weaker V-O-V bonds and form new V-O-Li bridges (Fig. 5(b))^26^. Also, there is a possibility to take place a redox reaction ($2{V}^{5+}+{O}^{2-}\to 2{V}^{4+}+{O}_{2}\uparrow$) during the melting of the batches at 900 °C. The V^5+^ ions take a part as a network forming positions with VO_5_ structural units which can be formed V-O-Li linkages^24^. Therefore, the influence of Li ions on the V=O bond is restricted and have an indirect manifestation which leads to preserve this band at ~1022 cm^−1^ ^26^. Thus, the V=O band is partially affected by Li_2_O doping in the present samples.

#### Raman spectroscopy analysis

**Figure 6:**
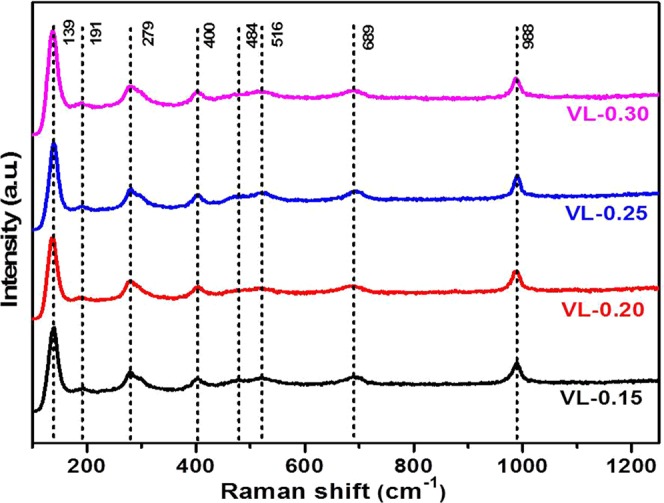
Raman spectra of Li_2_O doped vanadium oxide as quenched samples.

Raman spectra of as-quenched samples (Fig. 6) show the expected vanadia signals around 139, 191, 279, 400, 484, 516, 689 and 988 cm^−1^. The sharp Raman band at 988 cm^−1^ is attributed to the symmetric stretching of V = O groups in pure V_2_O_5_ ^29,30^. The intensity of this sharp V = O band increases with lithium content, establishes the formation of multilayer structures of V_2_O_5_ ^23^. The other Raman bands around 400 and 516 cm^−1^ are attributed to the existence of the characteristic layer structure of crystalline V_2_O_5_. Besides these, a small shoulder at 484 cm^−1^ is also observed, which can be due to the symmetric stretching of the V-O-V bonds in V_2_O_5_ ^29,30^. The band at 400 cm^−1^ can also be due to the Li-O stretching. Another band at 279 cm^−1^ is assigned to the deformational mode of the surface vanadyl groups. The other band around 689 cm^−1^ is due to the stretching vibration of oxygen ion in bridging position between three vanadia centers^29,30^. Finally, Raman band at 191 and 139 cm^−1^ are associated to the [VO_5_]-[VO_5_] vibrations and strongly accompanying with the layered structure. They are observed at low wavenumbers due to the heavy [VO_5_] units^31^. Presence of all these vibrations (Figs. 4–6) substantiate the structural changes and formation of multiphase lithium vanadate in the as-quenched samples as discussed in X-ray diffraction and FTIR analysis sections.

#### UV-visible spectroscopy analysis

**Figure 7:**
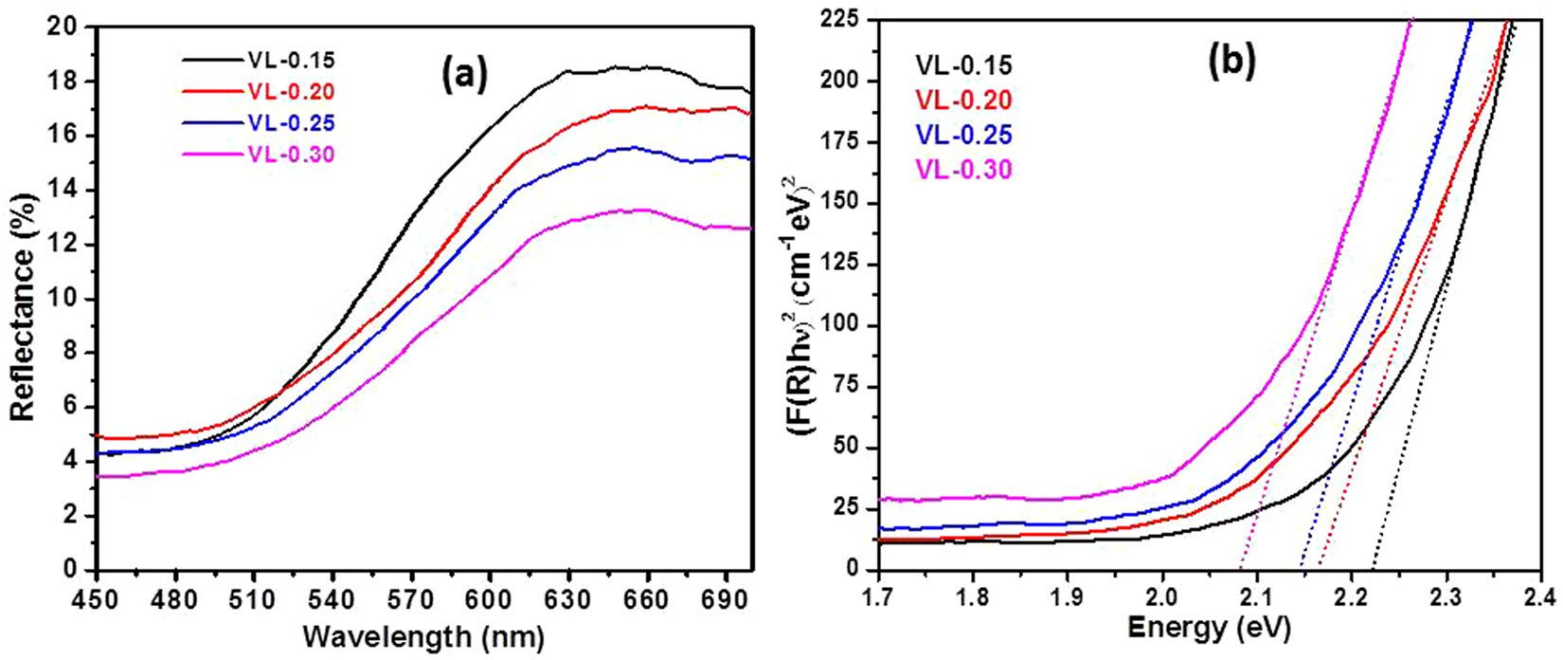
(**a**) The UV-vis spectra; and (**b**) the Tauc plots for V_2−x_Li_x_O_5−δ_ systems.

Figure 7(a) represents the UV-visible diffused reflectance spectra (DRS) as a function of wavelength for all the samples. The reflectance for VL-0.15, VL-0.20, VL0.25 and VL-0.30 samples are 18.5%, 17%, 15.6% and 13.2%, respectively. The quenched reflectivity could be ascribed to the optical phonon confinement, which leads to the light trapping in the samples^32^. This decrement in reflectivity with increasing Li_2_O dopant into V_2_O_5_ lattice can be due to the reflectance onset/absorption edge towards longer wavelengths (red shift). It is good agreement with other reports^32,33^. This reflectance data is used to calculate the optical band gap by Kubelka-Munk function^34^.

1 $$F(R)=K/S={(1-R)}^{2}/2R$$

where, *F*(*R*), *K* and *S* are the Kubelka-Munk function, absorption coefficient and scattering coefficient, respectively. The optical band gap is obtained from DRS using following equation.

2 $$(F(R)h\nu )=k{(h\nu -{E}_{g})}^{n}$$

Where, *k*, *E*_*g*_ and $h\nu$ are the proportionality constant, optical band gap and incident photon energy, respectively. The exponent ‘*n*’ depends on the transition, where n = 1/2, 2, 3/2 and 3 for allowed direct, allowed indirect, forbidden direct and forbidden indirect transition, respectively^34^. The direct optical band gap of all the samples is obtained using the Tauc plot (Fig. 7(b)) by extrapolating the straight-line portion of the plots of ${(F(R)h\nu )}^{2}$ versus $h\nu$ to energy axis (x-axis) at y = 0. The Urbach energy (*E*_*U*_) of the samples is calculated using the equation$\,\alpha (\nu )=\beta \exp (h\nu /{E}_{U})$. The inverse of the slope of the linear portion from *lnF*(*R*) versus $h\nu$ is used to measure the Urbach energy of the samples. The optical band gap decreases with increasing content of Li_2_O into V_2_O_5_ for all the samples. The optical band gaps of the present samples are in the semiconductor range (2.22–2.08 eV) and comparable to earlier reported values by another research group^35^. The sample with the lowest concentration of Li_2_O (VL-0.15) shows the highest optical band gap (2.22 eV) while the sample with the highest concentration of Li_2_O (VL-0.30) shows the lowest optical band gap (2.08 eV). It indicates that the Li_2_O concentration increases the defects in the present systems. The existence of variable oxidation states of vanadium as confirmed by FTIR, Raman spectroscopy and TG analysis (as discussed in next section) can be responsible for the creation of defects and oxygen vacancies in the present samples. In addition, there is a possibility to create new energy level above the valence band due to doping of Li_2_O. So, the filled valence band electrons easily excited to new generated energy level by dopant led to decrease the optical band gap of the samples^36^. The Urbach energy (*E*_*U*_) of the samples increases with increasing Li_2_O content (Table 2). VL-0.30 sample shows the highest *E*_*U*_ with the lowest optical band gap signifying the presence of higher disorder as compared to other samples^34^. The optical band gap and Urbach energy both are following opposite trend to each other. It indicates that defects increase with an increase in the doping of Li_2_O in place of V_2_O_5_.

#### Thermal properties

**Figure 8:**
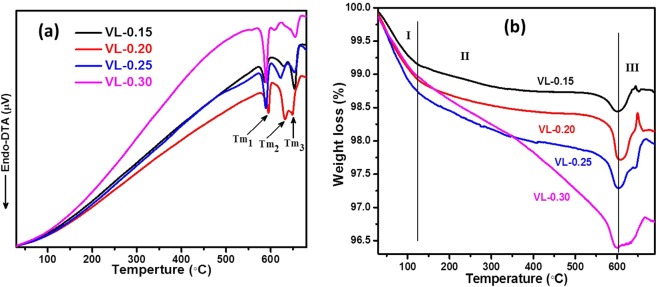
(**a**) DTA thermographs; and (**b**) thermal stability nature of Li_2_O modified V_2_O_5_ systems measured at 10 °C heating rate.

Figure 8(a) represents the DTA curves of lithium-doped V_2_O_5_ as-quenched samples in the temperature range of 30–800 °C. In DTA curves, all the samples exhibit three distinct endothermic peaks at a higher temperature (≥571 °C), which result from the melting points (Tm_1_, Tm_2_ and Tm_3_) of three different phases as observed in XRD patterns. Figure 8(b) shows the TG curves of the Li_2_O doped V_2_O_5_ as-quenched samples, which exhibit typical weight loss behavior. TGA thermograph shows a total weight loss of ~5% in the temperature range of 30–800 °C. The weight loss can be considered in three steps as marked I (30–120 °C), II (120–600 °C) and III (600–700 °C) in the TGA graph. The I step is assigned to the departure of adsorbed species and loss of water molecules^34^. The II step is associated to the reduction of V^5+^ to V^4+^/V^3+^ ions which can be shown by the following equation^24,34^.

3 $$2{V}^{5+}+{O}^{2-}\to 2{V}^{4+}+{O}_{2}\uparrow $$

In the III step, a little weight gain is observed in all the samples which can be related to the oxidation of V^3+^ to V^5+^ as written by the following equation^37,38^.

4 $${V}_{2}{O}_{3}+{O}_{2}={V}_{2}{O}_{5}$$

This weight gain during the oxidation at the higher temperature can be associated with the combination of O_2_ and its vacancies in the present samples. The reduction of these systems is expected due to vanadium is believed to have a mixed valence state (V^+3^, V^+4^ and V^5+^)^24,37,38^. It is also observed that weight loss increases with the addition of Li_2_O on the cost of V_2_O_5_ which leads to a decrease in the thermal stability of the present samples (I and II steps). It is manifestation of waterphilic nature of the samples and it increases with an increase in Li_2_O concentration. The reduction of vanadium from V^5+^ state to lower valance state is also increases with the addition of Li_2_O content. The chemical stability also decreases with Li_2_O concentration which is also manifested by Urbach energy and FTIR analysis of the samples. All the samples exhibit good thermal and chemical stability in the temperature range of ~300–550 °C without any phase transition (Fig. 8(a,b)) except VL-0.30 sample. However, sample with the lowest content of Li_2_O (7.5 mol%, VL0-0.15) shows the highest thermal and chemical stability in the temperature range of ~300–550 °C in comparison to other samples. Therefore, these samples can be used as electrolytes for solid oxide fuel cells or batteries applications.

#### Conducting studies

**Figure 9:**
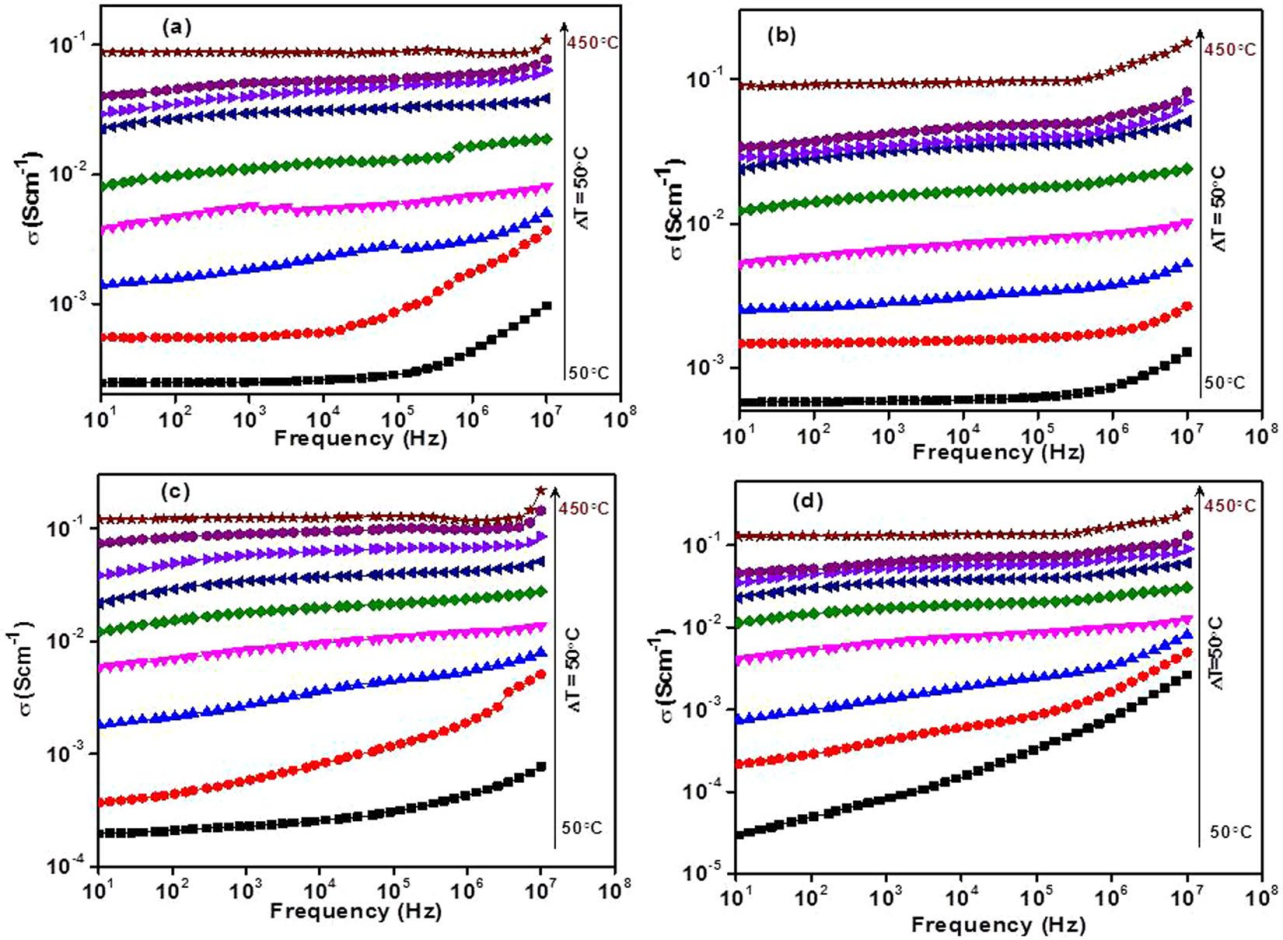
Change in conductivity as a function of frequency and temperatures for (**a**) VL-0.15 (**b**) VL-0.20 (**c**) VL-0.25 (**d**) VL-0.30 samples.

**Figure 10:**
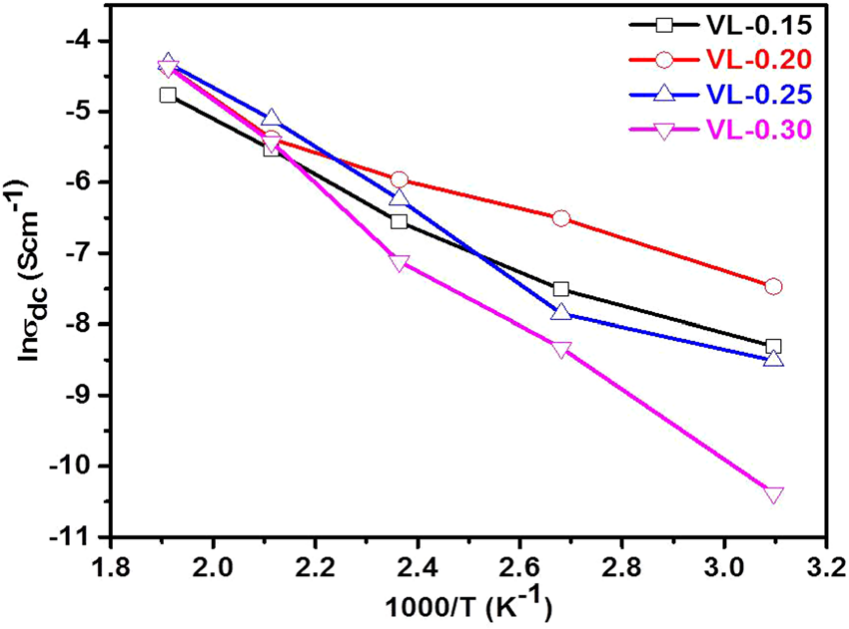
Change in dc conductivity as a function of the reciprocal temperature of VL-0.15 to VL-0.30 samples.

**Figure 11:**
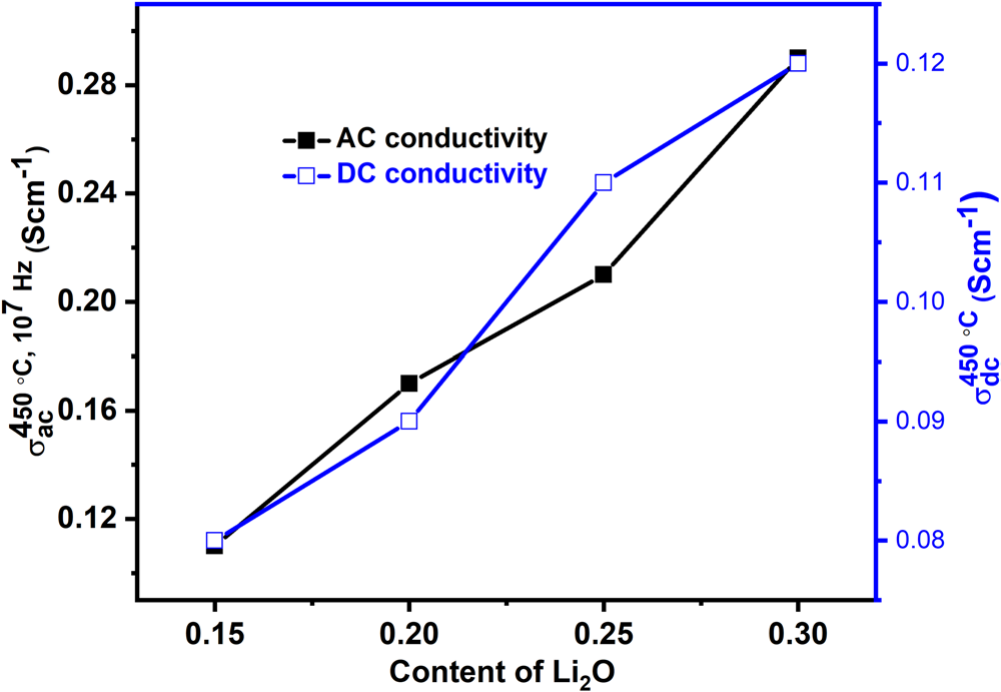
Change in AC and DC conductivity with Li_2_O content in V_2_O_5_ for all samples.

It is a well-known fact that hopping mechanism and band conduction are responsible for the electrical conduction in these types of materials^6,39^. The frequency dependent conductivity, *σ*_*ac*_ of the samples is determined using the following relation^6,39^.

5 $${\sigma }_{ac}(\omega )={\varepsilon }_{0}\omega ^{\prime\prime} =2\pi f\varepsilon ^{\prime} tan\delta $$

where, *ε*_0_ *ε*′, *ε*″, *f* and *tanδ* are the permittivity of free space (8.854 × 10^−12^ Fm^−1^), dielectric constant, dielectric loss, frequency (in Hz) of the applied electric field and tangent of loss, respectively. Figure 9(a–d) illustrates the AC conductivity (*σ*_*ac*_) variation with frequency and temperatures (50–450 °C, step size of 50 °C). The conductivity is almost frequency independent at low frequency region (plateau region) and corresponds to the DC conductivity (*σ*_*dc*_) of the samples^40^. At the same time, the conductivity is dependent on frequency *(σ*_*ac*_) at higher frequency region (dispersion region) and increases with increasing frequency^6,15,39,40^. The plateau region signifies the randomness of ionic diffusion which is due to the long-range order transport and appreciable diffusion of mobile ions in response to the applied ac field while the dispersion region signifies the conductivity relaxation phenomena in present samples^6,7,15,40,41^. It is also observed that *σ*_*ac*_ increases with increasing temperature (50–450 °C) for all the samples which supports the semiconductor nature of the samples^6,42^. Generally, the conductivity in such type of materials depends on the concentration and mobility of mobile ions/charge carriers i.e. electrons and holes^6,42^. The mobility of mobile ions/charge carriers increases with increasing temperature which leads to increasing overall conductivity as observed in the present samples. It can be understood by the fact that thermal energy (temperature) excites more electrons from valence band to conduction band resulting in higher conductivity. Further, according to the Jonscher’s universal power law, *σ*_*ac*_ can be written as^6,41,43,44^:

6 $${\sigma }_{ac}(\omega ,T)={\sigma }_{dc}(T)+A{\omega }^{s}$$

where, A is the temperature dependent constant of the material, ω is the angular frequency and *s* is the frequency and temperature dependent exponent that lies between 0–1. DC conductivity (*σ*_*dc*_(*T*)) is calculated to extrapolate AC conductivity at the lowest frequency (Fig. 9) and given in Table 2. The activation energy (*E*_*a*_) of the conduction is calculated using the Arrhenius plot (Fig. 10) between ln*σ*_*dc*_ and reciprocal of temperature (1000/*T*) by the following equation.

7 $${\sigma }_{dc}={\sigma }_{o}\exp (-{E}_{a}/{k}_{B}T)$$

where, *σ*_*o*_ and *k*_*B*_ are the pre-exponential factor, and Boltzmann’s constant, respectively. The values of *σ*_*ac*_ and *σ*_*dc*_ show an increasing trend with an increase in lithium content (7.5–15 mol%) as shown in Fig. 11. AC conductivity lies in the range of 0.11–0.29 Scm^−1^ at 450 °C and 10^7^ Hz whereas DC conductivity lies in the range of 0.08–0.12 Scm^−1^ at 450 °C. In vanadate materials, conductivity depends on the small polarons hopping (SPH) between V^4+^ site to the neighboring V^5+^ site^6,7,39^. SPH directly affects by the mobility and number of mobile charge carriers. On the replacement of V^5+^ by Li^+^, structure of the systems becomes more open due to transition from VO_4_ tetrahedral to VO_5_ trigonal bipyramid as discussed in physical parameters and structural properties. This structural change is responsible for a significant increase in oxygen vacancies with an increase in hopping of ions which leads to an increase in conductivity^4,5,42^. In addition, decrement in density, inter-ionic distance, polaron radius and optical band gap of the samples also support this increasing trend of conductivity with the addition of Li_2_O on the cost of V_2_O_5_ ^7^. The calculated activation energy values of dc conduction are depicted in Table 2. The activation energy increases with increasing content of Li_2_O except VL-0.20 sample and lies in the range of 0.26–0.43 eV (Table 2). These activation energy values clearly suggest that the ionic conduction increases with Li_2_O concentration into V_2_O_5_ in the present samples.

## Conclusion

The influence of Li_2_O addition on physical parameters, structural, optical, thermal and conducting properties of V_2−*x*_Li_*x*_O_5−*δ*_ (0.15 ≤ *x* ≤ 0.30) systems have been investigated. XRD patterns confirmed the presence of three crystalline phases. The presence of mixed valence states of vanadium is confirmed by spectroscopic investigations. The optical band gap is decreased with Li_2_O concentration. Thermal stability is found to decrease with the Li_2_O addition in place of V_2_O_5_. AC and DC conductivity are found to enhance by the addition of Li_2_O due to the creation of more hoping sites. Sample with the highest content of Li_2_O (15 mol%, VL-0.30) exhibited the highest DC conductivity i.e. 0.12 Scm^−1^ at 450 °C. It is observed that the transport mechanism in lithium vanadate systems is attributed to small polaron hopping (SPH) between V^4+^ to V^5+^ states, which is increased by the addition of lithium into vanadium. The present samples exhibit good DC and AC conductivity. Therefore, these materials can be considered as electrolytes for batteries/solid oxide fuel cells applications.

## Materials and Methods

Powder of V_2_O_5_ (Loba, Chemie, 99% purity) and Li_2_O (Sigma Aldrich, 97% purity) were used as raw materials to prepare compositions of V_2−x_Li_x_O_5−δ_, where x = 0.15(VL-15), x = 0.20(VL-20), x = 0.25(VL-25) and x = 0.30(VL-30) via melt and quench technique. The prescribed compositions are mixed using agate mortar and pestle in acetone medium for 2 h. The mixed batches were melted at 900 °C in recrystallized aluminum crucibles in an electric furnace followed by copper plates quenching in the air. The standard Archimedes principle is used to measure density (*D*) at room temperature of as-quenched samples with xylene as immersion liquid (0.863 gcm^−3^). The molar volume (*V*_*m*_) of as-quenched samples was calculated using the following equation,

8 $${V}_{m}=\sum _{i}{M}_{i}/\rho $$

where, *ρ* and *M*_*i*_ are the density and molar mass of the samples, respectively. The concentration of Li ions (*N*) was calculated by$\,{N}_{i}={N}_{A}\times mol \% \,of\,cation\times valency/{V}_{m}$, where *N*_*A*_ and *M* is the Avogadro’s number and molecular weight, respectively. The average inter-ionic distance (*R*_*i*_) was calculated using$\,{R}_{i}={(1/N)}^{1/3}$. The polaron radius was calculated by$\,{r}_{p}=({R}_{i}/2)\times {(\pi /6)}^{1/3}$. The X-ray diffraction (XRD) patterns of crushed samples were recorded by PANalytical X’Perts Pro MPD diffractometer with Cu-Kα radiations (λ = 1.54 Å). The scan rate and scan range were 2°min^−1^ and 10–80°, respectively. Fourier transform infra-red (FTIR) spectra of as-quenched samples were recorded in the wavenumber range of 200–4000 cm^−1^ at room temperature using Perkin Elmer-Spectrum-RF-1 FTIR spectrometer. The finely ground powder of as-quenched sample and KBr powder are mixed together to palletized for the FTIR measurement. The Raman spectra of the powder samples were recorded over the spectral range of 100–5000 cm^−1^ using Features STR 500 Airix Raman system equipped with Ar laser (532 nm) as the excitation source.

A double beam UV-Vis spectrophotometer (Model: Hitachi 3900H) was used to record diffused reflectance spectra (DRS) at room temperature between the wavelength 200–800 nm. Thermal analysis was carried out using TG/DTA (Exstar TG/DTA 6300) instrument of as-quenched powder samples in N_2_ medium with a heating rate of 10 °C/min over the range of room temperature to 1000 °C. The dielectric and conductivity analysis carried out using SI-1260 Solartron analytical LCR impedance analyzer. Both sides of the as-quenched samples are coated with Pt using 3000-FC auto fine coater from JEOL to serve as an electrode. The variation in impedance with frequency and temperature were studied from 10Hz-1MHz and 50–400 °C, respectively.
